# Reduced antiretroviral drug efficacy and concentration in HIV-infected microglia contributes to viral persistence in brain

**DOI:** 10.1186/s12977-017-0370-5

**Published:** 2017-10-16

**Authors:** Eugene L. Asahchop, Oussama Meziane, Manmeet K. Mamik, Wing F. Chan, William G. Branton, Lothar Resch, M. John Gill, Elie Haddad, Jean V. Guimond, Mark A. Wainberg, Glen B. Baker, Eric A. Cohen, Christopher Power

**Affiliations:** 1 Department of Medicine (Neurology), University of Alberta, Edmonton, AB Canada; 2 Department of Psychiatry, University of Alberta, Edmonton, AB Canada; 3 Department of Pathology, University of Calgary, Calgary, AB Canada; 4 Department of Medicine, University of Calgary, Calgary, AB Canada; 5 CHU Sainte-Justine, Montréal, Canada; 6 Department of Pediatrics, Université de Montréal, Montréal, Canada; 7 Department of Microbiology, Infectiology and Immunology, Université de Montréal, Montréal, Canada; 8 Montreal Clinical Research Institute, Montréal, Canada; 9 CIUSSS du Centre-Sud-de-l’ile-Montréal, CLSC des Faubourgs, Montréal, QC Canada; 10 McGill University AIDS Centre, Lady Davis Institute for Medical Research, Jewish General Hospital, Montréal, QC Canada

**Keywords:** HIV-1, Nervous system, Antiretroviral therapy, BLT mouse, Macrophages, Microglia

## Abstract

**Background:**

In patients with HIV/AIDS receiving antiretroviral therapy (ART), HIV-1 persistence in brain tissue is a vital and unanswered question. HIV-1 infects and replicates in resident microglia and trafficking macrophages within the brain although the impact of individual ART drugs on viral infection within these brain myeloid cells is unknown. Herein, the effects of contemporary ART drugs were investigated using in vitro and in vivo models of HIV-1 brain infection.

**Results:**

The EC_50_ values for specific ART drugs in HIV-infected human microglia were significantly higher compared to bone marrow-derived macrophages and peripheral blood mononuclear cells. Intracellular ART drug concentrations in microglia were significantly lower than in human lymphocytes. In vivo brain concentrations of ART drugs in mice were 10 to 100-fold less in brain tissues compared with plasma and liver levels. In brain tissues from untreated HIV-infected BLT mice, HIV-encoded RNA, DNA and p24 were present in human leukocytes while ART eradicated viral RNA and DNA in both brain and plasma. Interruption of ART resulted in detectable viral RNA and DNA and increased human CD68 expression in brains of HIV-infected BLT mice. In aviremic HIV/AIDS patients receiving effective ART, brain tissues that were collected within hours of last ART dosing showed HIV-encoded RNA and DNA with associated neuroinflammatory responses.

**Conclusions:**

ART drugs show variable concentrations and efficacies in brain myeloid cells and tissues in drug-specific manner. Despite low drug concentrations in brain, experimental ART suppressed HIV-1 infection in brain although HIV/AIDS patients receiving effective ART had detectable HIV-1 in brain. These findings suggest that viral suppression in brain is feasible but new approaches to enhancing ART efficacy and concentrations in brain are required for sustained HIV-1 eradication from brain.

**Electronic supplementary material:**

The online version of this article (doi:10.1186/s12977-017-0370-5) contains supplementary material, which is available to authorized users.

## Background

From the outset of the HIV/AIDS epidemic, early entry and expression of HIV-1 in the brain and cerebrospinal fluid (CSF) has been consistently observed [1, 2]. With the availability of increasingly efficacious antiretroviral therapy (ART), plasma and CSF viral burden is usually undetectable by conventional assays [3]. The impact of ART on viral burden in brain tissue has been less certain because of the relative inaccessibility of tissue for measurements except at autopsy; indeed, patients with HIV/AIDS rarely die while receiving effective ART. Nonetheless, with ART-associated improvement in immune status, the severity of degenerative brain disease associated with HIV/AIDS, termed HIV-associated neurocognitive disorders (HAND), has declined although it remains a serious challenge in ~ 25% of treated patients that often precludes employment and reduces overall survival [4–7]. All immunosuppressive lentiviruses (e.g., Human, Simian, Feline and Bovine Immunodeficiency Viruses) infect the brain and display a preference for macrophage tropism as well as infection and depletion of T lymphocyte populations. In the human brain, productive HIV-1 infection is evident in trafficking macrophages (bone marrow-derived macrophages) and resident microglia, but few if any infected T cells are observed [8–11]. These myeloid cell populations in the brain constitute a unique reservoir in which HIV-1 replicates because of the paucity of resident lymphoid cells within the brain and the blood–brain barrier’s impervious structure, which limits drug penetration. Of note, microglia and macrophages are less permissive to HIV-1 than CD4+ T-cells, possibly due to reduced CD4 expression and variable expression of specific host restriction factors [12–14]. Furthermore, macrophage-lineage cells are long lived (half-life of months–years) and comparatively resistant to cell death upon HIV-1 infection compared to CD4+ T-cells, although some groups have reported very short half-lives in HIV-infected macrophage-lineage cells [15, 16]. Likewise, recent studies of cerebrospinal fluid (CSF) show higher than expected rates of viral escape in CSF that were associated with brain injury in the setting of undetectable virus in plasma [17].

The impact of contemporaneous ART drugs on viral expression and replication in the brain remains unclear despite their potent viral suppressive properties in blood. Likewise, the actual ART drug concentrations in brain tissue are also uncertain. Studies of brain tissues from HIV-infected humans or SIV-infected nonhuman primates show that both viral RNA and DNA are detected in brain tissues despite receiving ART [15, 18, 19]. Of note, a very recent study reported that in a macrophage-only mouse model of HIV-1 infection, HIV-infected monocyte-derived macrophages were highly responsive to ART although the brain myeloid cells and tissues were not examined [20]. In these patients and animal studies, the efficacy of ART was assessed in combination but not as individual drugs. These circumstances raise several questions regarding the efficacy of ART in HIV-infected brain myeloid cells, ART’s capacity to modulate HIV-1 entry of the brain and the impact of ART on established HIV-1 infection of the brain. It is important to evaluate the efficacy of individual ART ex vivo and in animal studies to select ART drugs with a high propensity to inhibit replication in the brain.

Given the questions concerning the impact of ART on HIV-1 brain infection, we investigated ART drugs’ efficacies and concentrations in different experimental platforms. The in vitro and in vivo efficacies and concentrations of different ART drugs were examined in a primary cell culture model. HIV-1 DNA and RNA quantities in brain and plasma from HIV-infected immunosuppressed mice reconstituted with human leukocytes (BLT) mice and human actively receiving ART was also quantified. These studies showed that several ART drugs exhibit reduced concentrations and efficacies in the brain myeloid cells (and in brain tissues) compared to PBMCs, but nonetheless inhibited in vivo HIV-1 brain infection in BLT mice. Investigation of brain tissues from HIV/AIDS patients indicated there was persistent viral detection in the setting of longstanding aviremia with concurrent ART.

## Results

### HIV-1 infection of microglia and macrophages

**Fig. 1:**
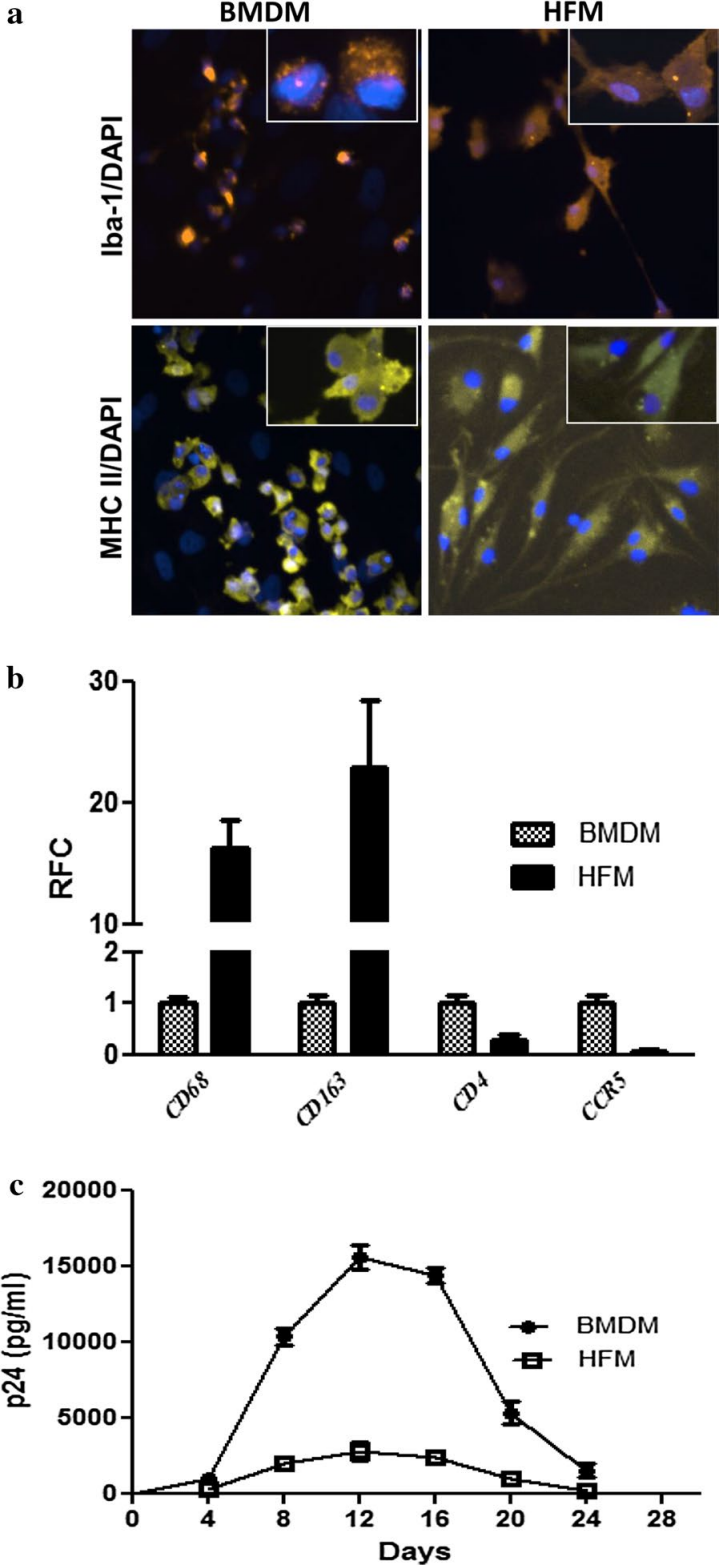
Human myeloid cells and HIV-1 infection. **a** Immunolabeling of cultured human fetal microglia (HFM) and bone marrow-derived macrophages (BMDMs) with antibodies to Iba-1 (HFM: orange, BMDMs: orange) and MHC Class II (HFM: yellow, BMDMs: yellow) expression. **b** Relative *CD68, CD163, CD4* and *CCR5* transcript expression levels in HFM and BMDMs were quantified by RT-PCR. **c** HFM and BMDMs were infected at a multiplicity of infection (MOI) of 0.1 with HIV-1 YU-2 (derived by transfection in 293 T-cells) and supernatant was collected after every 4 days for HIV-1 p24 quantification by ELISA

In the brain, HIV-1 infects both resident microglia and circulation-derived macrophages originating from the bone marrow although viral production tends to be higher in macrophages [1, 21–23]. To model HIV-1 infection of these brain myeloid cells, we established primary cultures of human fetal microglia (HFM) and bone marrow-derived macrophages (BMDMs). Following differentiation in vitro to enhance microglial and macrophage phenotypes, both cell types expressed Iba-1 and MHC Class II (Fig. 1a). HFM showed higher transcript levels of *CD68* and *CD163* but conversely BMDMs showed greater expression of *CD4* and *CCR5* (Fig. 1b). After HIV-1_YU2_ infection at matched input titers (MOI = 0.1), BMDMs showed increased release of HIV-1 p24 in supernatants over time compared to HFM, with peak p24 release at Day 12 post-infection (Fig. 1c). Thus, both BMDMs and HFM supported HIV-1 replication but BMDM exhibited higher viral production levels, possibly due to increased CD4 and CCR5 expression in keeping with previous observations suggesting there is higher viral replication in macrophages compared to microglia [23].

### ART efficacy in HIV-infected myeloid cells and PBMCs

**Fig. 2:**
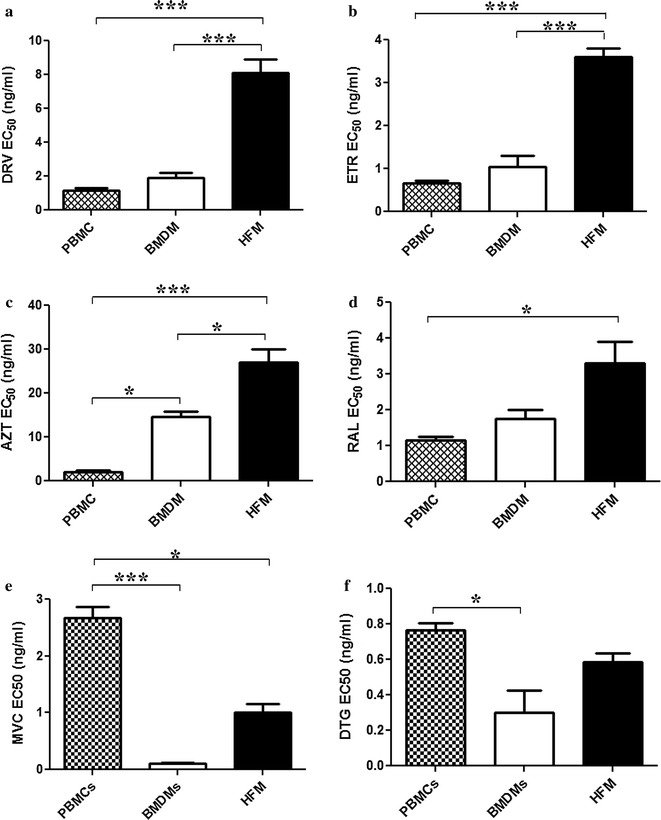
ART drug EC_50_ values in HIV-infected HFM, BMDMs and PBMCs. HFM, BMDMs and PBMCs were infected with HIV-1 YU-2 (MOI of 0.1), followed by drug treatment. Mean EC_50_ values for each cell type (HFM, BMDM and PBMC) are shown during treatment with **a** DRV, **b** ETR, **c** AZT, **d** RAL, **e** MVC and **f** DTG. * *p* < 0.05; ** *p* < 0.01; *** *p* < 0.001

Although the efficacies of different ART drugs in T cell lines and primary CD4 T cells is well known, little is known about their effects in HIV-infected brain myeloid cells although they suppress viral replication while their relative actions in different brain myeloid cells (such as microglia) versus activated PBMCs is unknown. To assess the comparative antiviral activity of different ART drugs in HFM, BMDMs and activated peripheral blood mononuclear cells (PBMCs), representative drugs from several different classes were investigated. These included zidovudine (AZT), etravirine (ETR), raltegravir (RAL) and darunavir (DRV), maraviroc (MVC) and dolutegravir (DTG). Each drug was tested in a concentration-dependent manner by measuring p24 levels in supernatants from HIV-infected HFM, BMDMs and activated PBMCs (Additional file 1) from which EC_50_ values were determined (Table 1). Comparison of ART drug efficacies in each infected cell culture type revealed that DRV (Fig. 2a), ETR (Fig. 2b), AZT (Fig. 2c) and RAL (Fig. 2d) had significantly higher EC_50_ values in HFM compared to activated PBMCs and in BMDMs for DRV (Fig. 2a), ETR (Fig. 2b) and AZT (Fig. 2c). In contrast, MVC and DTG were both highly effective at inhibiting viral replication in HIV-infected BMDMs and HFM compared to activated PBMCs (Fig. 2e, f). These findings underscored the differential actions of individual ART drugs on viral replication in brain myeloid cells.

**Table 1:** ART EC_50_ (ng/ml) values in different myeloid cells and PBMCs compared to published CSF concentrations

ART drug	PBMC EC_50_	BMDM EC_50_	HFM EC_50_	CSF^a^
AZT	2.1 ± 0.24	14.7 ± 1.2	27.0 ± 3.1	44.0 ± 6.0
RAL	1.1 ± 0.1	1.8 ± 0.2	3.3 ± 0.6	21.3 ± 5.0
ETR	0.64 ± 0.08	1.1 ± 0.25	3.6 ± 0.2	8.4 ± 1.1
DRV	1.2 ± 0.15	1.9 ± 0.3	8.1 ± 0.78	47.0 ± 8.8
MVC	2.67 ± 0.2	0.1 ± 0.02	1.0 ± 0.15	2.87 ± 0.4
DTG	0.77 ± 0.04	0.3 ± 0.13	0.59 ± 0.05	18

^a^All CSF concentrations are published data

**Fig. 3:**
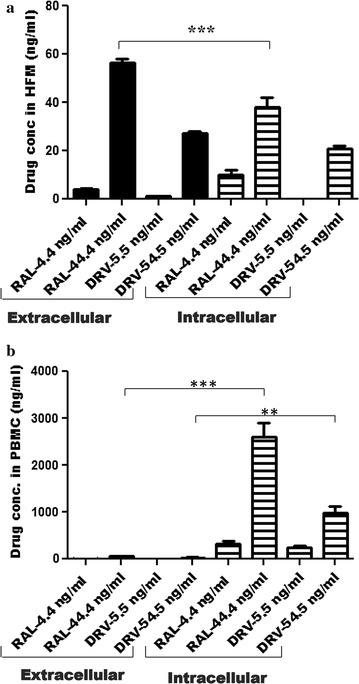
Intracellular and extracellular ART drug concentrations in HFM and PBMCs. HFM (**a**) and PBMCs (**b**) were exposed to DRV and RAL at different concentrations for 24 h. Intracellular and extracellular concentrations were measured by HPLC–MS. * *p* < 0.05; ** *p* < 0.01; *** *p* < 0.001

As ART concentrations within cells (and tissue) are critical determinants of their antiviral capacity, we measured DRV and RAL concentrations in cultured cells. In these analyses both RAL and DRV exhibited similar intracellular and extracellular concentrations in HFM apart from RAL, which at 44.4 ng/ml showed higher extracellular compared to intracellular concentrations (Fig. 3a). However, the intracellular concentrations for both drugs were significantly higher in activated PBMCs compared to HFM cultures (~ 100 fold) and matched extracellular activated PBMCs concentrations (Fig. 3b). Of note, exposure of differentiated human myeloid (THP-1) cells to endotoxin or HIV-1 infection did not influence RAL or DRV extra- or intracellular concentrations (Additional file 2). However, published antiretroviral (ARV) drug concentrations in cerebrospinal fluid (CSF) [24–36] were higher than the EC_50_ values for all drugs tested herein (Table 1). These data indicated that ART efficacy in productively infected brain (myeloid) cells was substantially less than in infected lymphoid cells for specific ART drugs.

### In vivo tissue ART drug concentrations

**Fig. 4:**
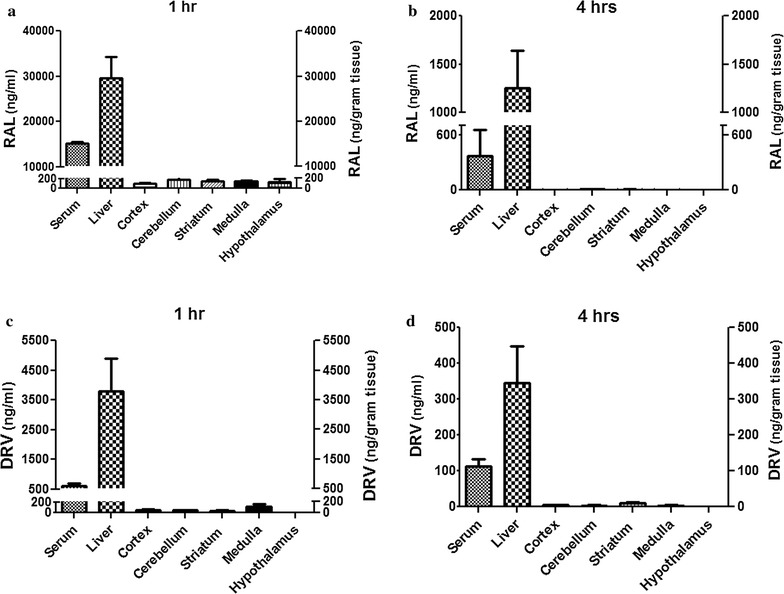
In vivo ART drug concentrations in brain, blood and liver. RAL and DRV were injected intraperitoneally into adult mice (1.2 mg/animal). ART drug concentrations were measured at 1 and 4 h post-drug administration in serum, liver and brain (cortex, striatum, cerebellum, medulla and hypothalamus). RAL was detected in all tissues at 1 h (**a**). At 4 h, **b** RAL levels were markedly reduced in serum and liver and not detected in brain. **c** DRV was detected in all tissues at 1 h but levels of DRV were also markedly reduced at 4 h (**d**). Results represent mean ± SEM (n = 4)

Although ART drug concentrations in CSF are known in treated HIV/AIDS patients [24–36], brain tissue levels are largely unknown for most ART drugs, prompting investigation of RAL and DRV in vivo concentrations. Following intraperitoneal administration of each drug in adult mice (1.2 mg/animal), drug concentrations were measured at different time points in serum, liver and the brain (cerebral cortex, striatum, cerebellum, medulla and hypothalamus). When RAL was administered, the mean concentrations in serum and liver were approximately 15,000 ng/ml and 27,000 ng/g tissue, respectively, at 1 h post-administration while the concentration in different regions of the brain ranged between 150 and 200 ng/g of tissue (Fig. 4a). At 4 h post-administration the serum concentrations for RAL in serum and liver were 300 ng/ml and 1200 ng/g, respectively, with only traces of RAL in brain (Fig. 4b). For DRV, there was a similar trend with lower drug concentrations in brain; in the serum and liver, the mean DRV concentrations were 850 ng/ml and 3800 ng/g tissue, respectively, at 1 h post drug administration (Fig. 4c). However, in different brain regions, DRV concentrations ranged between 0 and 150 ng/g (tissue) after 1 h (Fig. 4c). Again, only trace amounts of DRV were detected at 4 h post-administration (Fig. 4d). These studies showed that while ART drug levels were similar in different anatomic regions of the brain, they were substantially lower than their corresponding concentrations in serum or liver.

### In vivo efficacy of ART in HIV-infected humanized mice

**Fig. 5:**
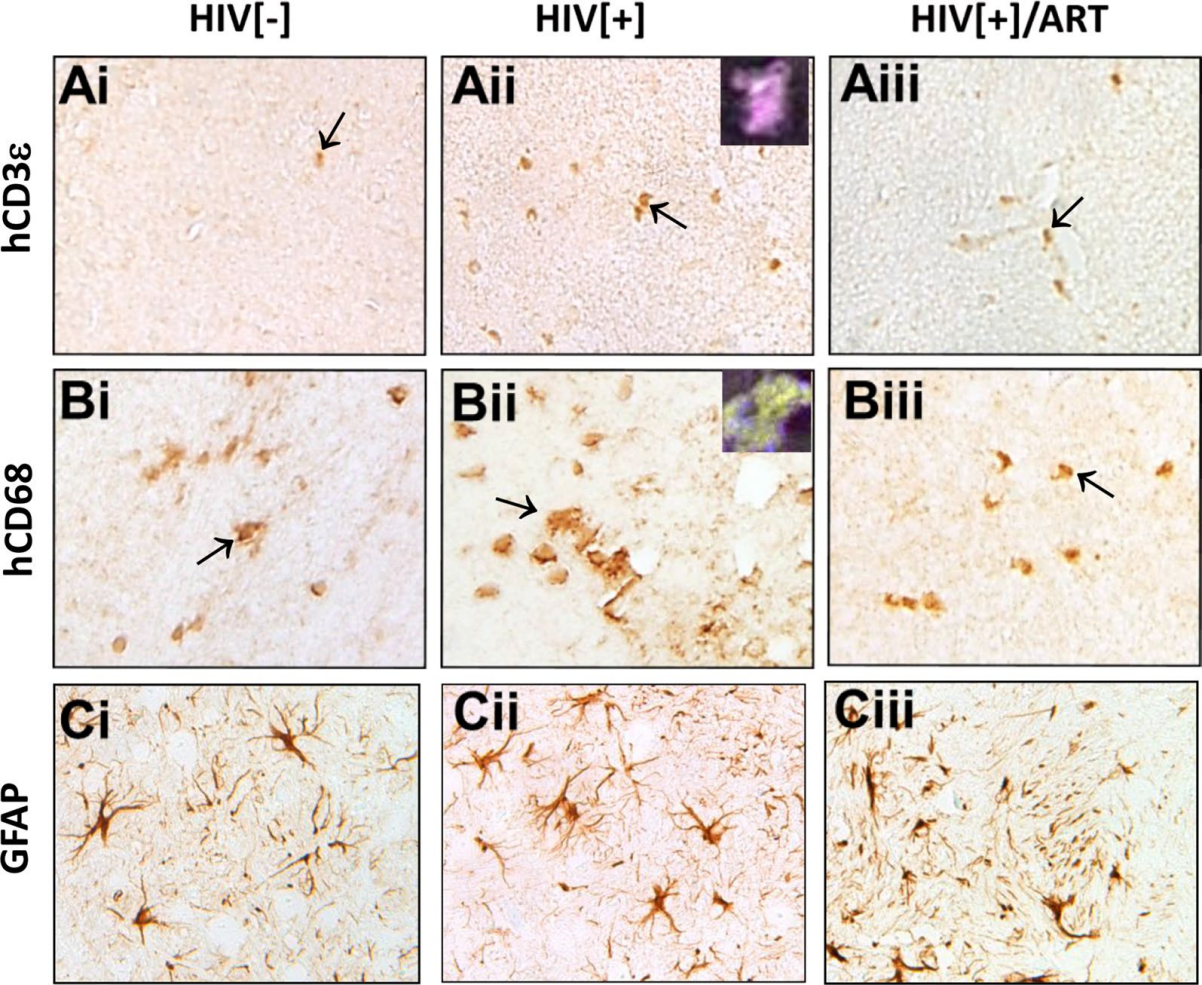
Neuropathology in HIV-infected BLT mice: Human CD3ε immunolabeled T cells were observed in brains from uninfected (ai), HIV-infected (aii) and HIV-infected/ART-exposed animals (aiii). Co-labeled cells exhibiting CD3ε (yellow), p24 (purple) with DAPI nuclear labeling (inset) were evident in HIV-infected animals. Similarly, human CD68 immunolabeling of macrophages was evident in HIV[−] (bi), HIV[+] (bii) and HIV[+]/ART (biii) and was localized showing CD68 (yellow) and HIV-1 p24 (purple) (inset). GFAP-immunopositive (murine) astrocytes were apparent in brains from HIV[−] (ci), HIV[+] (cii) and HIV[+]/ART (ciii) animals

As contemporary ART regimens are highly effective in controlling viral burden in blood, we wanted to examine if there was a corresponding inhibition of HIV-1 infection in the brain during viral suppression in blood. To address this issue, we evaluated uninfected and HIV-infected humanized (BLT) mice with and without an ART regimen comprised of RAL, tenofovir and emtricabine or PBS (Fig. 5). The neuropathological features associated with HIV-1 infection of the brains of BLT mice were assessed in brain sections from animals in each group using antibodies to conventional neural cell markers that immunolabeled human CD68 (macrophage/microglia), GFAP (astrocytes), CD3ε (lymphocytes) and HIV-1 p24. Human CD3ε immunopositive T cells were detected in HIV[−], HIV[+] and HIV[+]/ART (Fig. 5ai–iii) animals with p24 immunoreactivity evident within CD3ε^+^ cells (Fig. 5aii inset). Similarly, human CD68 immunopositive macrophages were detected in brains from each experimental group, i.e. HIV[−], HIV[+] and HIV[+]/ART (Fig. 5bi–iii) animals, also with p24 immunodetection in CD68^+^ cells (Fig. 5bii, inset). GFAP-immunolabeled (mouse only) astrocytes were apparent in brains from all experimental groups with similar morphology and abundance in all groups (Fig. 5ci–iii). Human cells engrafted mouse brains in all experimental groups without substantial differences in host (mouse) glial responses despite the presence of HIV-1 p24 in the untreated/infected experimental group. Thus, HIV-1 infection of BLT resulted in viral neuroinvasion mediated by infected macrophages and T cells but ART treatment of infected animals lead to undetectable virus in the brain without preventing human cell trafficking into the brain.

**Fig. 6:**
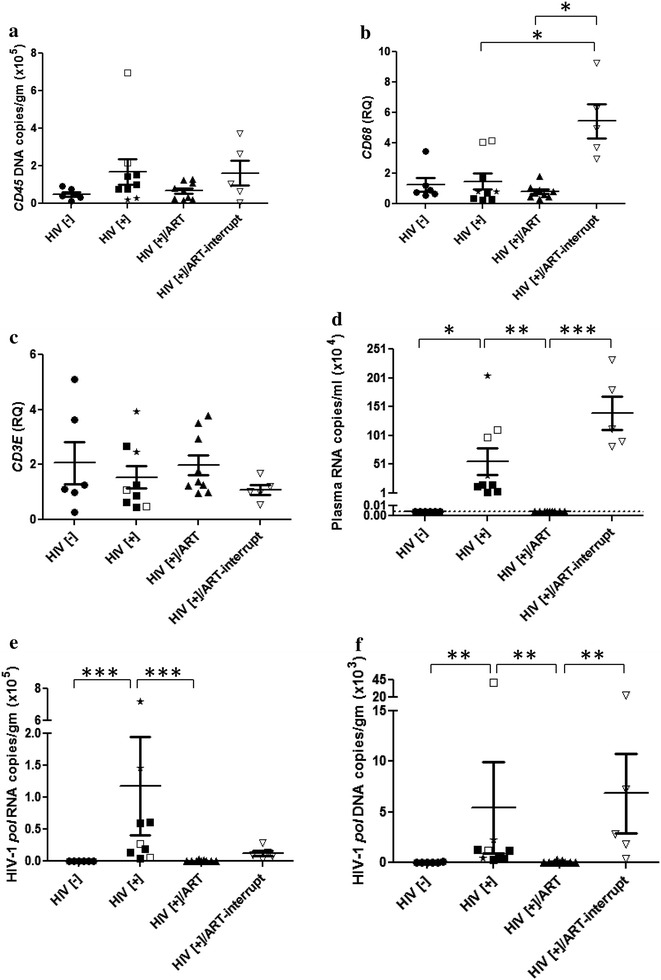
HIV-1 and human RNA and DNA in brains from BLT mice. Four experimental groups of BLT mice were examined including uninfected (HIV[−]), HIV-infected/PBS-treated (HIV[+]), HIV-infected/ART-treated (HIV[+]/ART) and HIV-infected/ART-interrupted (HIV[+]/ART-interrupt). The mice in the HIV[+] group were infected at different TCID_50_/animal as well as for different durations [two mice were infected at 50,000 TCID_50_/animal for 8 weeks without viral detection and then re-infected at 50,000 TCID_50_/animal for another 4 weeks (asterisk); two other mice were infected at 50,000 TCID_50_/animal for 13 weeks (open square); five mice were infected at 100,000 TCID_50_/animal for 7 weeks (filled square)]. For the ART-interrupt group (HIV[+]/ART-interrupt), animals were infected and treated for 2 weeks (between weeks 4–6 or 8–10), after which ART was stopped and animals were sacrificed at 13 weeks post-infection. **a** Human *CD45* DNA (copy numbers/g of tissue) was detected in all animals’ brains and did not differ across experimental groups. **b** Human *CD68* transcript was also detected in all animals but increased in the HIV[+]/ART-interrupt group. **c** Human *CD3E* was detected in all experimental groups. **d** HIV-1 RNA was detected in plasma in the HIV[+] and HIV[+]/ART-interrupt groups. **e** All brains from the HIV[+] and HIV[+]/ART-interrupt groups showed detectable HIV-1 RNA. **f** HIV-1 DNA was detected in brains from the HIV[+] and HIV[+]/ART-interrupt groups. * *p* < 0.05; ** *p* < 0.01; *** *p* < 0.001

By way of verifying the above neuropathological findings, we examined viral, human and mouse gene expression levels in brains from each experimental BLT mouse group together with examining the impact of interrupting ART for several weeks to recapitulate clinical circumstances of missed ART doses. To determine the relative number of human cells engrafting each brain, human *CD45* genomic DNA copy numbers were analyzed in brain tissues and were found to be similar across all experimental groups (Fig. 6a). Human *CD68* transcript levels in brain varied across experimental groups, with increased *CD68* expression in the brains of the HIV[+]/ART-interrupt group (Fig. 6b) while *CD3E* transcript levels were similar across groups (Fig. 6c). Analyses of human immune gene expression in brains showed that *CD163* (Additional file 3A) was detectable but did not differ across groups although IP10 (*CXCL10)* and *MX2* (Additional file 3B and C) were significantly increased in the HIV[+]/ART-interrupt group. Of note, the mouse macrophage gene, *f4/80,* was significantly decreased in the HIV[+]/ART-interrupt group (Additional file 3D).

Viral burden was examined next, disclosing that plasma mean HIV-1 RNA copy number in HIV-infected BLT mice ranged widely and was detected in the HIV[+] group (520,000 copy numbers/ml) and in the HIV[+]/ART-interrupt group (1,150,000 copy numbers/ml) (Fig. 6d) but was not detected in the HIV[+]/ART or HIV[-] groups. In brains from BLT mice, mean viral RNA levels ranged from 500 to 700,000 copies/g (tissue) in the HIV[+]/PBS group but HIV-1 RNA was not detectable in the HIV[−] or the HIV[+]/ART groups (Fig. 6e). In contrast, mean HIV-1 RNA copy numbers in the HIV[+]/ART-interrupt group were detectable in all animals but at low copy numbers (100–500 copies/g) (Fig. 6e). Total mean HIV-1 DNA copies were also measured in all groups, revealing that the HIV[+] group showed 50–40,000 copies/g but HIV-1 DNA was not detected in the HIV[−] or the HIV[+]/ART groups (Fig. 6f). As with HIV-1 RNA brain burden, mean HIV-1 DNA copy numbers in the HIV[+]/ART-interrupt group were detectable in all animals at 100–20,000 copies/g (Fig. 6f) although integrated proviral DNA in brain was not detected in all groups. Of note, RAL was not detected in the brains of the ART-treated animals. These findings highlighted the capacity for HIV-1 to infect brain myeloid cells but also emphasized that in HIV[+]/ART group viral RNA was undetectable in the brain.

### HIV-1 detection and neuroimmune responses in human brain tissue during effective ART

**Fig. 7:**
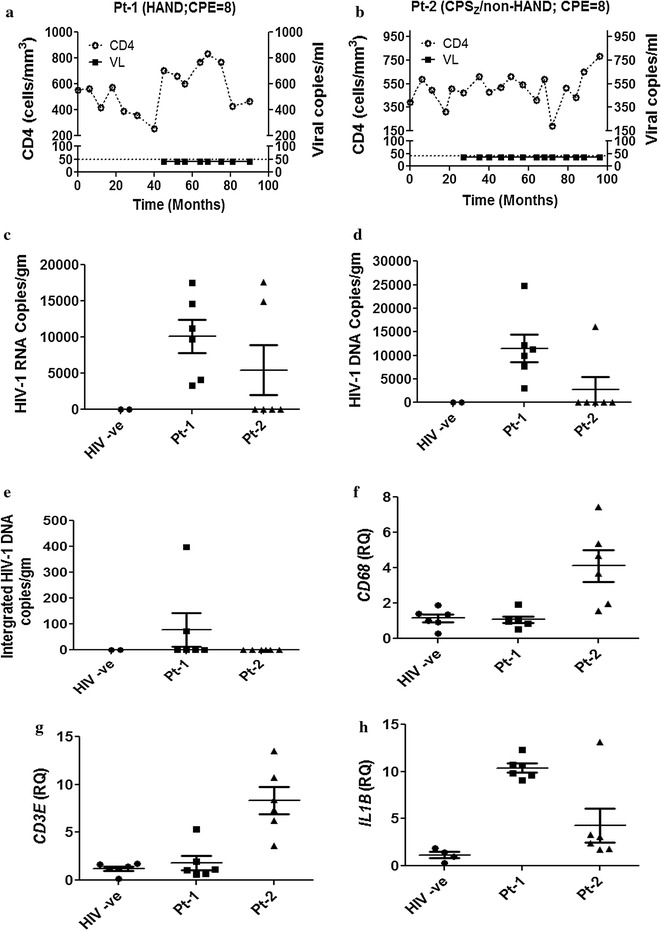
HIV-1 RNA, DNA and host gene expression in human brain during ART. HIV-infected patients receiving effective ART were followed for > 90 months before brain tissue was obtained by autopsy (Pt-1) (**a**) or by surgical resection for epilepsy (Pt-2) (**b**). **c** Viral RNA and **d** total viral DNA were quantified by droplet digital PCR in brain tissues from uninfected patients (HIV − ve) and from Pt-1 and Pt-2. **e** Integrated proviral DNA was measured in brain by real-time PCR using specific primers and probe to HIV-1 *LTR* following pre-amplification with an *alu*-*gag* primer pair. **f** *CD68*, **g** *CD3E* and **h** *IL1B* transcript levels were measured in brain tissue by RT-PCR. Results represent mean of ± SEM

To date, most, if not all, reported studies of HIV-1 quantities in brain were performed using tissues from patients who had died days to weeks after last ART dosing, precluding a definitive comparison between virus burden in brain tissue and plasma during effective ART. We encountered two patients with HIV/AIDS from whom brain tissue was collected within hours of last ART dosing. Patient 1 (Pt-1) had a premortem diagnosis of HAND but died unexpectedly following a myocardial infarction while Patient 2 (Pt-2) had longstanding complex partial epilepsy (CPS_Z_) and underwent surgical resection of the left temporal lobe to remove the epileptic lesion (see “Methods” section for clinical and demographic details). Pt-1 (Fig. 7a) and Pt-2 (Fig. 7b) were receiving effective ART regimens associated with high CNS penetration effectiveness (CPE) scores within 8 and 12 h, respectively, of brain tissue collection. Both patients had CD4 T cell levels of > 400 cells/μl and plasma RNA viral loads below the detection limit (< 40 copies/ml) for at least three previous years of clinical follow up. In multiple discrete brain samples (n = 6) from HIV-infected and non-infected (control) patients including cortex and adjacent white matter, HIV-1 RNA and DNA as well as host immune responses were measured (Fig. 7c–f and Additional file 4). All 6 brain samples from Pt-1 contained HIV-1 RNA while two of 6 samples from Pt-2 had detectable HIV-1 RNA. The brain samples from uninfected patients did not show HIV-1 RNA (Fig. 7c). Total HIV-1 DNA was also quantified from matched samples, revealing that in Pt-1 viral DNA was detected in all 6 samples but in Pt-2 only one brain sample showed viral DNA, while in uninfected controls (n = 6), viral DNA was not detected (Fig. 7d). Using an established assay that detected virus in HIV-infected human macrophages (Additional file 4A), integrated proviral DNA was measured, revealing that integrated provirus was detected in 2 of 6 brain samples from Pt-1 but was not detected in brain samples from Pt-2 (Fig. 7e). To determine the corresponding expression of cellular and immune genes in ART-suppressed HIV/AIDS patients’ brains compared with uninfected patients, we examined *CD68* (macrophage marker), *CD3E* (T cell marker), *CD163*, *TNFA, IL6* and *IL1B* transcript levels; *CD68* (Fig. 7f), *CD3E* (Fig. 7g) and *CD163* (Additional file 4B) were increased in samples from Pt-2 compared to Pt-1 and uninfected controls. In contrast, *IL6* and *TNFA* were each increased in Pt-1 and Pt-2, respectively (Additional file 4C and D), while *IL1B* expression was increased in both HIV/AIDS patients’ brains compared to uninfected controls (Fig. 7h). Similarly, *APOBEC3G, MX2, MX1* and *BST2,* were upregulated in Pt-2’s brain but not induced in Pt-1’s brain samples (Additional file 4E–H). Using tissue from six different regions of the brain from Pt-1, the mean RAL concentrations (18 ng/g tissue) were low (Additional file 5). These data indicated that HIV-1 RNA and DNA with associated neuroinflammatory responses were present in brain tissues from patients who had achieved long-term suppression of HIV-1 in plasma although concurrent ART drug levels were low.

## Discussion

The present study addresses several fundamental questions regarding the impact of ART on HIV-1 infection of the brain. To the best of our knowledge, this is the first report showing that contemporary ART drugs exert differential actions in HIV-infected brain myeloid cells depending on the infected cell type and individual drug, with overall higher EC_50_ values in HIV-infected microglia compared to macrophages and activated PBMCs. Additionally, this is the initial report comparing ART drug concentrations in human brain myeloid and activated PBMCs as well as in brain; the intracellular drug levels were substantially lower in microglia than in activated PBMCs and very low (and transient) in brain tissues from mice and humans. These studies also indicate that in ART-treated BLT mice, HIV-1 infection was undetectable within the brain (and plasma) but ART interruption resulted in a viral rebound. Our clinical observations supported the experimental findings of reduced efficacy in brain myeloid cells by disclosing that HIV-1 RNA and DNA were detected in brain tissues from stable aviremic HIV/AIDS patients receiving ART within hours of tissue collection. These findings underline the limited ART drug efficacies and concentrations in the brain together with the precarious vulnerability of the brain to HIV-1 infection derived from blood.

The human brain is a unique HIV-1 reservoir because it is established early in infection, harbors CCR5-dependent HIV-1 viruses, and the cell populations supporting HIV-1 replication are comprised solely of myeloid cells including trafficking macrophages and resident microglia [37]. Although in vitro data show that HIV-1 infects and replicates in both macrophages and microglia [21, 38, 39], data reporting replication of HIV-1 in human brain myeloid cells in the setting of ART-mediated viral suppression in blood are limited and usually complicated by interruption of ART before tissue collection [40, 41]. In SIV-infected macaques, infected macrophages and perhaps microglia contribute to viral persistence in the brain [18, 42]. Effective ART resulted in complete suppression of plasma SIV levels associated with undetectable SIV RNA in brain while viral DNA levels remain unchanged [18, 42]. A quantitative outgrowth assay was reported that assessed the contribution of productively infected microglia/macrophages in ART-suppressed SIV-infected macaques and found productively infected macrophage-lineage cells in different tissues including the brain [8].

Although integrated proviruses might exhibit defective replication [43], their capacity to exert pathogenic effects could be maintained through expression of subgenomic mRNAs encoding pathogenic viral proteins including Vpr, Tat and Nef. To date, there are few studies quantifying integrated proviral (HIV-1) DNA in human brain in the presence or absence of ART; the ratio of integrated:total HIV-1 DNA in brain ranged from 1:6 to 1–81 [11] in an early study, similar to that reported (1:86) for infected lymphocytes [44]. Another study reported matched integrated and unintegrated HIV-1 LTR sequences from the same brain were similar, but LTR activity was not assessed [45]; nonetheless, LTRs from replication-competent brain-derived HIV-1 exhibit substantial variation in transcriptional activity [46, 47]. Herein, the ratio of integrated:total viral DNA in ART-treated HIV/AIDS patients was 1:147 for Pt-1 but integrated provirus was not detected in brain samples from Pt-2 (Fig. 7e). While brain tissue from Pt-1 was collected from frontal cortex and proximal white matter, Pt-2’s brain samples were from temporal lobe, an anatomic site that typically shows less viral burden [48], which may have contributed to the relative paucity of virus in Pt-2. Another differentiating aspect between the two patients that could explain high viral burden in Pt-1 compared to Pt-2 is the composition of the ART regimen at the time of tissue collection. Pt-1 was receiving abacavir, lamivudine and raltegravir while Pt-2 was on abacavir, lavimudine and dolutegravir. We observed increased antiviral efficacy of dolutegravir in brain myeloid cells compared to activated PBMCs, implying a potentially higher intracellular concentration and perhaps greater antiviral activity for longer periods, as suggested by others [49]. As our in vitro data showed comparable efficacy in HFM and BMDMs with dolutegravir, its use in Pt-2 might explain the reduced viral burden and the absence of integrated virus.

While the ART drugs we tested (DRV, RAL, ETR, AZT, MVC, DTG) are highly effective in controlling HIV-1 replication in blood, the extent to which they regulate viral replication in HIV-infected brain myeloid cells is largely unknown. Indeed, their levels in tissues are also uncertain although they are known to enter CSF which has prompted assignment of individual CPE values. Previous studies have reported that several ART drugs are efficacious in HIV-infected monocyte-derived macrophages [50] and some also inhibit HIV-transduced astrocytes [51]. However, the current data indicate that, at matched input viral titers and drug concentrations, most of the ART drugs were significantly less efficient in controlling viral replication in HIV-infected microglia compared to BMDMs or activated PBMCs (Fig. 2a–d), although both MVC and DTG were highly efficacious. The increased efficacy of MVC is supported by studies indicating that intensification of ART in both HIV-1 and SIV infections with MVC improves neurocognition and further reduces latent viral reservoir in the brain [52–55]. It is probable that this feature of MVC likely stems from its capacity to block CCR5 as well as induce PPAR-gamma expression [56]. It has previously been shown that PPAR-gamma up regulation can inhibit HIV-1 replication [57]. Notably, activated PBMCs also appeared to concentrate RAL and DRV at higher intracellular levels compared to intracellular concentrations in microglia (Fig. 3a, b). Increased intracellular concentrations would enhance antiretroviral drug efficacy over time, leading to restricted viral integration and production. Our results also suggest that brain tissue is less accessible to intraperitoneally delivered drugs; ART drug concentrations were similar in different brain regions but substantially lower than in liver or blood (Fig. 4a–d), with minimal drug detection by 4 h post-delivery. Two independent studies have shown in lymphoid tissues of HIV/AIDS patients receiving ART with undetectable plasma viremia that there is continuous evolution and trafficking of HIV-1 persistent virus as well as lower tissue ART drug concentrations than in the peripheral blood [58, 59]. These findings also illustrate that viral persistence is associated with lower ART drug concentrations in lymphoid tissues. Collectively, the present data underline the limited efficacy of present-day ART drugs in the brain and perhaps other tissues/organs harboring HIV-infected tissue macrophages. The use of sub-optimal ART regimens might lead to low drug concentrations in infected brain cells, resulting in a viral reservoir in these long-lived cells together with the potential emergence of drug resistance.

We used a BLT murine model of HIV-1 infection [60] to examine the brain viral burden in animals infected with high titer HIV-1 and their responses to an established ART regimen. The present data are similar to a recent study [60] indicating that viral RNA (Fig. 6c) and total DNA (Fig. 6d) were detected in brains from BLT mice, although our data show higher viral levels, perhaps due to a higher input viral titer or efficiency of human cell engraftment. Nonetheless, viral RNA was undetectable in brain by the ART regimen implemented in this study in all animals treated until sacrifice while in animals in which ART was interrupted before termination of the study, both viral RNA and DNA were detected in brain, which was associated with an increase in human *CD68* expression. Remarkably, other than *CD68* expression, human (and mouse) host cell abundance and immune responses in BLT brains (Fig. 4) were minimally affected despite variable infectious input titers and durations of infection, unlike in human brains (Fig. 5).

## Conclusions

These findings highlight the complex circumstances involved in HIV-1 infection of the brain while also emphasizing the challenges in establishing brain-specific contemporary ART regimens. Our in vitro data are supported by data from human brain tissue showing that the efficacy of contemporary ART drugs is less in the brain compared to peripheral blood. This reduced efficacy might contribute to HIV-1 viral persistence in the brain. The higher in vitro efficacies of MVC and DTG in brain myeloid cells suggest that inclusion of these drugs in first line ART regimens might prevent or diminish the development of HIV-associated neurological impairments as well as viral persistence in the brain.

## Methods

### Patients

Brain tissue from two HIV-1 seropositive patients receiving ART without detectable viremia (Patients 1 and 2) and uninfected (n = 6) seronegative patients were obtained by autopsy or biopsy. HIV-infected patients were receiving ART as part of the active care program at Southern Alberta Clinic, Calgary, Alberta, Canada [4–7] while uninfected patients died of other causes. Brain tissue was collected from HIV-infected patients (Patient 1: autopsy following death from a myocardial infarction; Patient 2; surgical resection of left temporal lobe for chronic complex partial epilepsy and from adult uninfected Other Disease Control patients (n = 6; causes of death included sepsis [n = 1], cancer [n = 2], Parkinson’s Disease [n = 1], pneumonia [n = 2]) at autopsy after receipt of consent. Patient 1 (Pt-1, Caucasian male, 57 yr, HIV-1 B clade seropositive for 27 yr, lifetime CD4 T cell nadir = 78 cells/μl, disease course complicated by distal sensory neuropathy and HIV-associated neurocognitive disorders [HAND, MND subtype for 10 years]) at the time of death was receiving RAL, abacavir (ABA) and lamivudine (3TC) while Patient 2 (Pt-2, Caucasian male, 38 yr, HIV-1 B clade seropositive for 12 yr, lifetime CD4 T cell nadir = 386 cells/μl, complicated by complex partial epilepsy since childhood with normal cognition and no HIV-associated complications) at the time of surgery was receiving ABA, 3TC and DTG.

### CNS penetration effectiveness values of ART drugs

We used the updated CNS penetration effectiveness (CPE) values as reported previously [61]. The CPE value for DTG has not been established. However, based on a study on the ratio CSF to plasma concentration of DTG and as well as the CSF and plasma HIV-1 RNA viral load [36], we assumed a CPE value of 3 for this study.

### Generation of BLT mice, infection, ART and collection of brain tissue

BLT mice were generated and infected using sub-lethally irradiated NOD/SCID/IL2Rγ^−/−^ mice [obtained from Jackson Laboratory (Bal Harbor ME), 6-10 weeks old], by implantation of human fetal thymus under the kidney capsule and IV injection of fetal liver CD34+ cells [62]. After transplantation mice were monitored for engraftment of human cells by flow cytometry. The HIV-1 NL4-3 *env*_ADA_-IRES-GFP construct was used for virus production. Brain tissue (forebrain) and plasma were harvested from four groups of animals including uninfected (HIV[–]; n = 5), HIV-infected with PBS treatment (HIV[+]; n = 9), HIV-infected with ART treatment (HIV[+]/ART; n = 9) and HIV-infected that were treated with ART but interrupted before sacrifice (HIV[+]/ART-interrupt; n = 5). This latter group of mice was infected and treated for 2 weeks post-infection (between 4 and 6 or 8 and 10) and after 2 weeks of treatment, ART was stopped and the virus allowed to rebound and mice were sacrificed at week 13 post-infection. Animals in the (HIV[+]; n = 9) were infected for different durations or at different input titers (two mice that did not show infection after 8 weeks and were re-infected at a TCID_50_ 50,000/animal for another 4 weeks, another two mice were infected at a TCID_50_ 50,000/animal for 13 weeks and the other five mice were infected at a TCID_50_ 100,000/animal for 7 weeks, by intraperitoneal infection. HIV-infected animals were treated with ART (RAL, emtricitabine and tenofovir) or PBS for 3 weeks and sacrificed at week 7 post infection. In another group, mice were infected for 8 weeks, treated with the same ART regimen or PBS for weeks and sacrificed at 12 weeks post infection. Brains were harvested at the time of necropsy and a half hemisphere was fixed in 4% paraformadehyde (PFA) and the other half stored at − 80 °C. Plasma HIV RNA was quantified in 25 μl of plasma. In brief, 50 μl of plasma was diluted to 2 ml with normal human plasma; 1 ml of diluted plasma was used to quantify RNA copy numbers using the Abbott real time HIV-1 assay (sensitivity, < 40 copies/ml).

### RNA and DNA preparation

Total RNA and DNA were extracted from brain of BLT mice and HIV-infected and non-infected patients using the RNeasy and DNeasy kits (Qiagen Germantown MD, USA), respectively, according to manufacturer’s instructions and as previously described [18, 63].

### Quantitative real time PCR

First strand cDNA synthesis was performed using DNase-treated RNA, random primers and Superscript II reverse transcriptase (Invitrogen, Carlsbad, CA, USA) at 42 °C for 90 min according to the manufacturer’s instructions. cDNA was diluted 1:3 (adding 100 μl of ultrapure water to 50 μl of cDNA). Host human genes were quantified as previously described [63]. The sequences of primers used for quantification of host immune genes are included (Table 2).

**Table 2:** List of primers used for quantitative real time PCR

Primer	Sequence
hu_CD3E-F	5′-GCCTCCGCCATCTTAGTAAAG-3′
hu_CD3E-R	5′-TCTTCATTACCATCTTGCCCC-3′
hu_CD163-F	5′-GAGTCCCTTCACCATTACTGTG-3′
hu_CD163-R	5′-GACTTTCACTTCCACTCTCCC-3′
hu_IL1B-F	5′-CCAAAGAAGAAGATGGAAAAGC-3′
hu_IL1B-R	5′-GGTGCTGATGTACCAGTTGGG-3′
hu_IL6-F	5′-ACCCCTGACCCAACCACAAAT-3′
hu_IL6-R	5′-AGCTGCGCAGAATGAGATGAG-3′
hu_APOBEC3G-F	5′-CGAAGTGAAAACAAAGGGTCC-3′
hu_APOBEC3G-R	5′-CATACTCCTGGTCACGATGC-3′
hu_MX2-F	5′-AGCAGGAGATCACAAACAGG-3′
hu_MX2-R	5′-GGTAAGTCTTTCTGCCAGTCG-3′
hu_CD68-F	5′-CATCTCTGTACTGAACCCCAAC-3′
hu_CD68-R	5′-CCATGTAGCTCAGGTAGACAAC-3′
hu_TNF alpha-F	5′-GTTTGAATTCTTAGTGGTTGCC-3′
hu_TNF alpha-R	5′-ATTCAGGAATGTGTGGCCTGC-3′
hu_GAPDH-F	5′-ACCAGGGCTGCTTTTAACTC-3′
hu_GAPDH-R	5′-TTGATTTTGGAGGGATCTCG-3′
mouse_f4/80-F	5′-GCTGTGAGATTGAAGCA-3′
mouse_f4/80-R	5′-AGTTTGCCATCCGGTTACAG-3′
mouse_GAPDH-F	5′-AATGGTGAAGGTCGGTGTG-3′
mouse_GAPDH-R	5′-GTGGAGTCATACTGGAACATGTAG-3′

### Droplet digital PCR for viral RNA and DNA quantification

HIV-1 viral RNA was quantified using 5 μl of diluted cDNA as template. For genomic DNA (gDNA) quantification, 5 μl (200 ng) of DNA was incubated with the restriction enzyme Alu I for 5 min at room temperature together with the reaction mixture. The Bio-Rad QX200 droplet digital PCR system was used for quantification [57, 64]. For both RNA and DNA quantification the probe: 5′-/56-FAM/AAGCCAGGA/ZEN/ATGGATGGCC/3IABkFQ/-3′, primer 1: 5′-CAAATTTCTACTAATGCTTTTATTTTTTC-3′ and primer 2: 5′-GCACTTTAAATTTTCCCATTAGTCCTA-3′ were used. Human CD45 copy number in the brain of BLT mice was also quantified by droplet digital PCR. The probe: 5′-/5HEX/AGAATGTTC/ZEN/TGGCCCCTCAGTGC/3IABkFQ/-3′ and primer 1: 5′-GAAAAGCTCCCTGAAGCAAAG-3′, primer 2: 5′-GGTTTGGAGTTTCCTCATTTATGTC-3′. Droplets were generated from each reaction and consisted of 10 μl droplet digital PCR probe Supermix, 900 nM primers, 250 nM probe and template cDNA or gDNA in a final volume of 20 μl using a droplet generator. PCR was performed using an S1000 thermal cycler (Bio-Rad). The cycling conditions were as follows: 10 min at 95 °C, 30 s denaturation at 94 °C, 58 °C extension for 60 s, and 10 min at 98 °C for a total of 40 cycles. After the cycling, droplets were analyzed immediately or stored at 4 °C overnight and until analysis. Raw data were analyzed with the specific software (QX200) by setting a common fixed fluorescence threshold intensity based on non-template control (NTC). The number of template copies was then estimated based on the number of positives detected in a corresponding channel and the number of total accepted droplets. Samples were quantified in duplicate and the analysis repeated two or three times. Template copies per sample were calculated averaging the overall replicate wells per sample. We estimated the total number of cells from the concentration of gDNA input as described previously [64].

### Quantification of HIV-1 integration by real time PCR

A two step PCR amplification was performed using DNA extracted from tissues as template. The primers, probes and cycling conditions described in a previous study were used [65]. A serial dilution of U1 cells with a known concentration of DNA was also included and used to generate standard curve for quantification.

### Cell cultures, virus infections and ART drugs

For in vitro ART studies, HFM, BMDMs or adult PBMCs were infected and allowed to replicate in the presence or absence of ART drugs [38, 57]. Primary HFM were isolated from 16 to 20 week fetal brain tissues, as previously described [38]. Briefly, fetal brain tissues were dissected, meninges were removed, and a single cell suspension was prepared through enzymatic digestion with 0.25% trypsin and 0.2 mg/ml DNase I for 60 min at 37 °C in a water bath. This was followed by passage through a 70-μm cell strainer. The cells were washed twice and plated in vented Cell+ T-75 tissue culture flasks (Sarstedt) and cultures were incubated at 5% CO_2_ for two weeks. Cultures were maintained in MEM supplemented with 10% FBS, 2 mM l-glutamine, 1 mM sodium pyruvate, 1 × MEM nonessential amino acids, 0.1% dextrose, 100 U/ml Penicillin, 100 μg/ml streptomycin. After 2 weeks, cultures were gently rocked for 10 min to suspend the weakly adhering microglia in medium, which were then decanted, washed and plated. Purity of cultures was verified by immunofluorescence [38]. BMDMs were isolated from 16 to 20 week fetal femurs. Briefly, using a 5 ml syringe each femur was drilled and flushed with DMEM into a tube. The cell suspension was then incubated with ACK lysis buffer to remove red blood cells, washed and centrifuged twice. The cell pellets were re-suspended in DMEM (10% FBS) containing 25 ng/ml of macrophage colony stimulating factor (M-CSF), transferred into a six well culture plate and incubated at 37 °C and 10% CO_2_. Cell culture medium was replaced every 3 days and macrophages were harvested on Day 7. Human THP-1 cells were cultured in RPMI (10% FBS) and differentiated for 72 h with PMA (50 nM) [66]. After exposure to PMA, cells were washed with PBS and fresh medium without PMA was added and incubation continued for another 48 h before use. PBMCs were isolated from healthy donors using the Ficoll-Paque (Sigma) density gradient separation, stimulated with phytohemagglutinin-P (Sigma) for 3 days at 5 μg/ml and re-suspended at 3.5 × 10^6^ cells/ml in RPMI 1640 supplemented with 10% heat-inactivated FBS, 1% penicillin/streptomycin, 1% glutamine and 1% recombinant human IL-2 at which time cells were infected. Viruses were produced by transfection of 16 μg of HIV-1 YU-2 plasmid into HEK293T cells using Lipofectamine 2000 (Invitrogen, Carlsbad, CA). Two days after transfection, supernatants from transfected cells were harvested and centrifuged at 1500 rpm for 10 min to remove cellular debris, filtered through a 0.22-μm-pore-size filter, and stored in aliquots at − 80 °C. Virus production was confirmed by HIV-1 p24 ELISA [38]. For in vitro ART studies, HFM or BMDMs (5 × 10^4^) were seeded into 96-well plates. Plates were incubated at 37 °C and 5% CO_2_ overnight to allow cells to adhere to the bottom of the plate and infected the following day for 4 h at 37° C and 5% CO_2_ at a multiplicity of infection (MOI) of 0.1 for 4 h and washed (×3). For HIV-1 replication, supernatant was collected at pre-defined time points post-infection. A total of 250 μl of diluted drug at different concentrations was added to each well post-infection and incubated for 5 days. On Day 5 post-infection, supernatants were collected for HIV-1 p24 quantification by ELISA. For PBMCs infections, cells were infected with HIV-1 YU-2 for 2 h (MOI 0.05), washed and re-suspended with fresh medium and 125 μl of cell suspension containing 250,000 cells were seeded into each well of a 96-well plate. Diluted drugs (125 μl) at different concentrations were added to each well post-infection. On day 4 post-infection supernatant was collected for HIV-1 p24 quantification.

AZT, ETR, RAL and DRV were obtained from the NIH AIDS Research and Reference Reagent Program. Additional RAL and DRV used for animal studies were purchased (Cedarlane or Sigma respectively).

### Immunofluorescence and immunocytochemistry studies

Cultured fetal HFM, BMDMs and fixed brains were immunolabeled with specific antibodies as described previously [57, 67]. Briefly, fetal HFM and BMDMs were seeded into an 8-well chamber µ-slide (Ibidi, DE). Cells were fixed (4% PFA) for 20 min. and immunolabeled with antibodies to Iba-1 (rabbit pAb 1:250, Wako) or MHC II (Mouse mAb, 1:300, Dako) over night. After washes with PBS, cells were incubated with anti-rabbit (AlexaFluor 647 nm, red) and anti-mouse (AlexaFluor 488 nm, green) (1:250, Life Technologies) antibodies. Cell nuclei were stained with DAPI (1:100, ImmunoChemistry Technologies) for 15 min. An Olympus IX-81 confocal microscope (Quorum Technologies) was used to capture the images. Fixed brains from uninfected [–] and HIV-infected [+] BLT mice were paraffin-embedded followed by sectioning and mounting. Slides were rehydrated and subjected to antigen retrieval by boiling in 10 mM sodium citrate, pH6 before immunostaining. Tissues were labeled with primary antibodies [anti-hCD68 (Abcam), −hCD3ε (Millipore), anti-HIV-1 p24 (NIH AIDS reagents #530) and GFAP (Dako)] followed by application of appropriate secondary antibodies [57].

### ART concentration measurements

RAL and DRV were injected intraperitoneally into adult mice (8–12 months old) at a concentration of 1.2 mg per animal in a total of 4 animals per group and the subsequent drug concentrations were measured in blood, liver and brain. Animals were sacrificed at 1, 4, 8 or 24 h post-drug administration and whole blood, liver and brain (cortex, striatum, cerebellum, medulla and hypothalamus) were harvested and the concentrations of RAL and DRV were measured using HPLC–MS after homogenizing in 80% methanol, centrifuging, and running the supernatants through Oasis HLB Prime uelution plates followed by elution of the drugs. A similar procedure was used for analysis of RAL concentrations in human brain.

### Statistical analyses

One-way ANOVA (Kruskal–Wallis test) with multiple comparisons was used to analyze in vitro and in vivo experiments. Statistical tests were applied using GraphPad Prism version 5 (GraphPad Software, San Diego California USA, www.graphpad.com). Results are expressed as mean ± SEM. *p* values < 0.05 were considered significant.

## Additional files

**Additional file 1.** Representative dose-response curves for ART drugs (RAL, AZT and DRV measured by p24 ELISA in supernatant from PBMCs (**A**, **D** and **G**), BMDM (**B**, **E** and **H**) and HFM (**C**, **F** and **I**).

**Additional file 2.** Intracellular and extracellular ARV drug concentrations in differentiated human THP-1 cells that were stimulated with LPS or infected with HIV-1 and treated with DRV or RAL at different concentrations for 24 hours. Drug concentrations were measured by HPLC-MS.

**Additional file 3**. Brain tissue (70–100 mg) from all BLT HIV-infected and uninfected animals was used to extract RNA. The RNA was used to quantify host genes (**A**) human *CD163* (**B**) human *IP10* (**C**) human *MX2* and (**D**) mouse *f4/80*.

**Additional file 4.** Differentiated THP-1 cells were infected with HIV-1 YU-2 and SF162. DNA was extracted from infected cells and used to establish the integration assay (**A**). Host genes expression from patients 1 and 2 compared to uninfected controls measured by quantitative RT-PCR for, (**B**) *CD163*, (**C**) *IL6*, (**D**) *TNFA*, (**E**) *APOBEC3G*, (**F**) *MX2*, (**G**) *MX1* and (H) *BST2*.

**Additional file 5.** Brain tissue (70–100 mg) from different anatomic regions from patient 1 and uninfected controls were used to measure RAL concentration by HPLC-MS.
