# Supplementary figures and images for: Reduced antiretroviral drug efficacy and concentration in HIV-infected microglia contributes to viral persistence in brain
Source: Retrovirology. 2017 Oct 16;14:47. doi: 10.1186/s12977-017-0370-5 (PMC5644262; doi:10.1186/s12977-017-0370-5)

**A**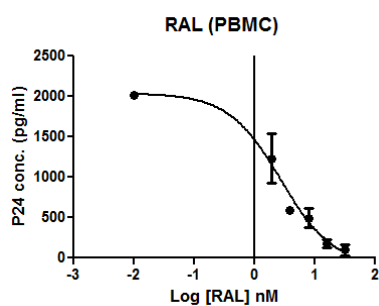**B**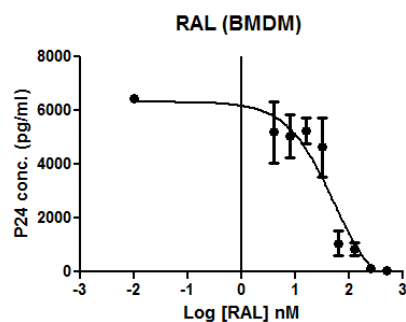**C**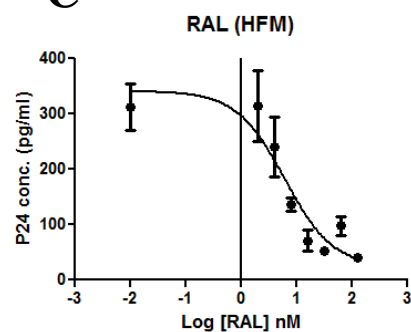**D**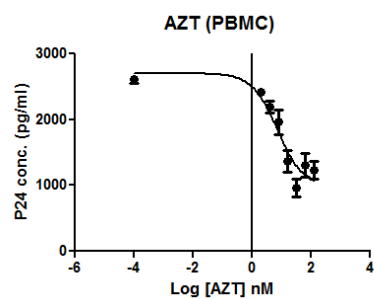**E**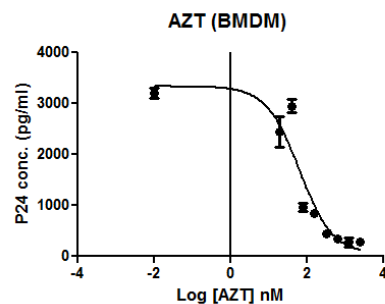**F**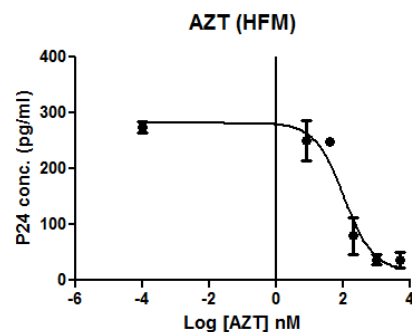**G**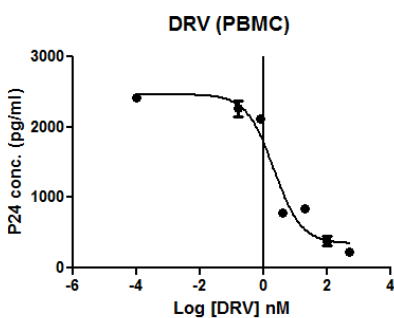**H**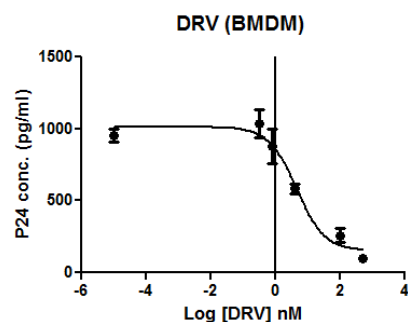**I**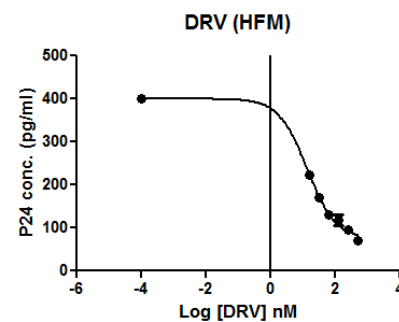

Supplement: Supplementary file 1 — Additional file 1. Representative dose-response curves for ART drugs (RAL, AZT and DRV measured by p24 ELISA in supernatant from PBMCs (A, D and G), BMDM (B, E and H) and HFM (C, F and I). [file 12977_2017_370_MOESM1_ESM.pdf]

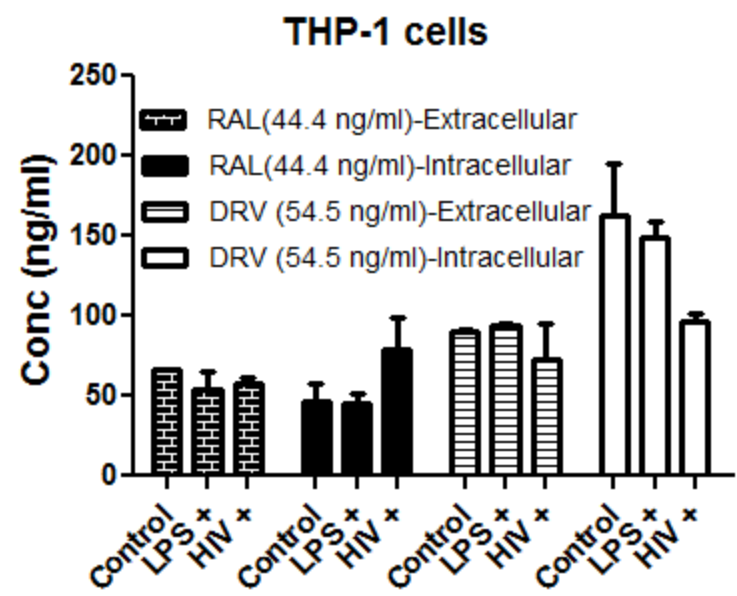

Supplement: Supplementary file 2 — Additional file 2. Intracellular and extracellular ARV drug concentrations in differentiated human THP-1 cells that were stimulated with LPS or infected with HIV-1 and treated with DRV or RAL at different concentrations for 24 hours. Drug concentrations were measured by HPLC-MS. [file 12977_2017_370_MOESM2_ESM.pdf]

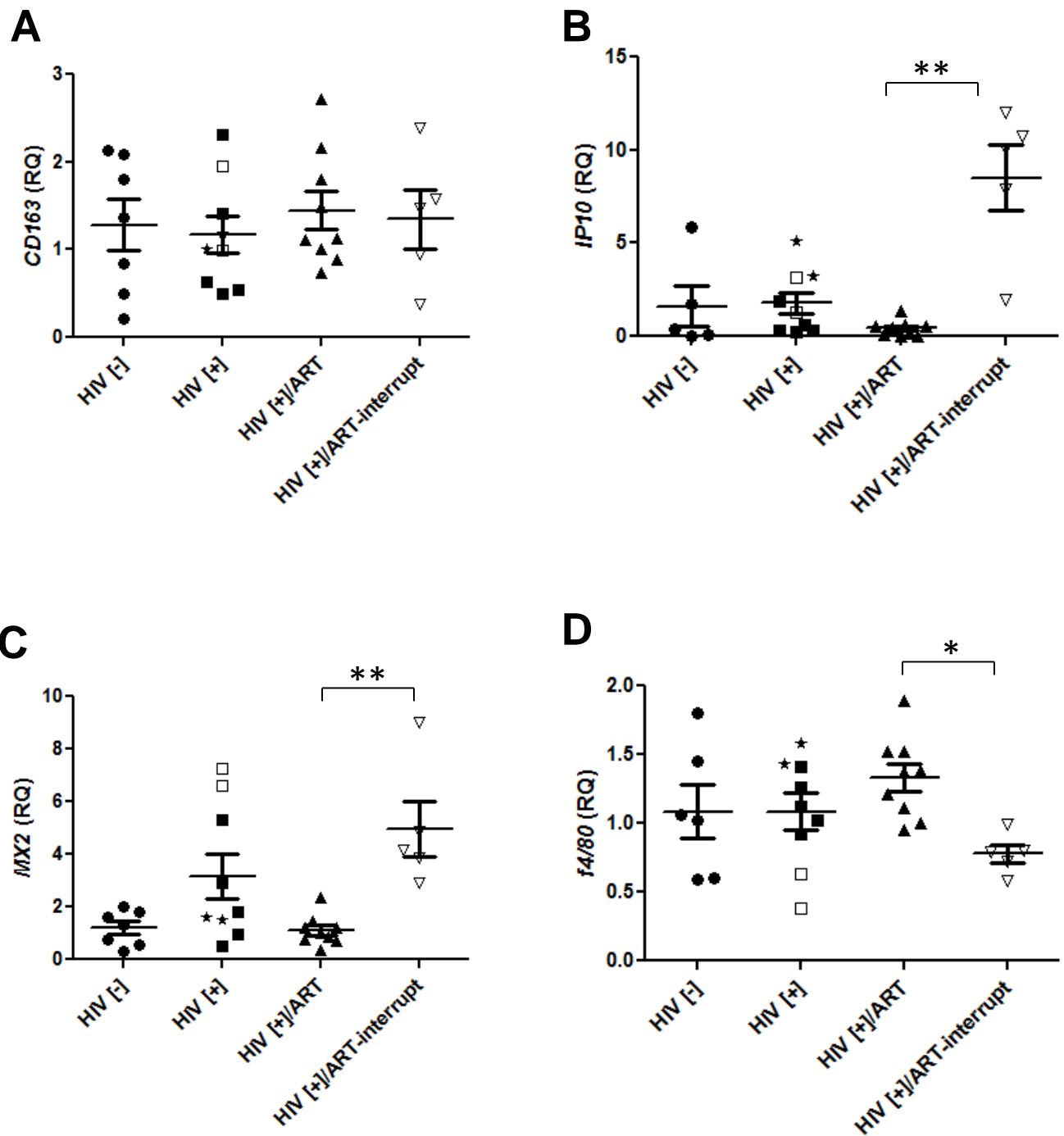

Supplement: Supplementary file 3 — Additional file 3. Brain tissue (70–100 mg) from all BLT HIV-infected and uninfected animals was used to extract RNA. The RNA was used to quantify host genes (A) human CD163 (B) human IP10 (C) human MX2 and (D) mouse f4/80. [file 12977_2017_370_MOESM3_ESM.pdf]

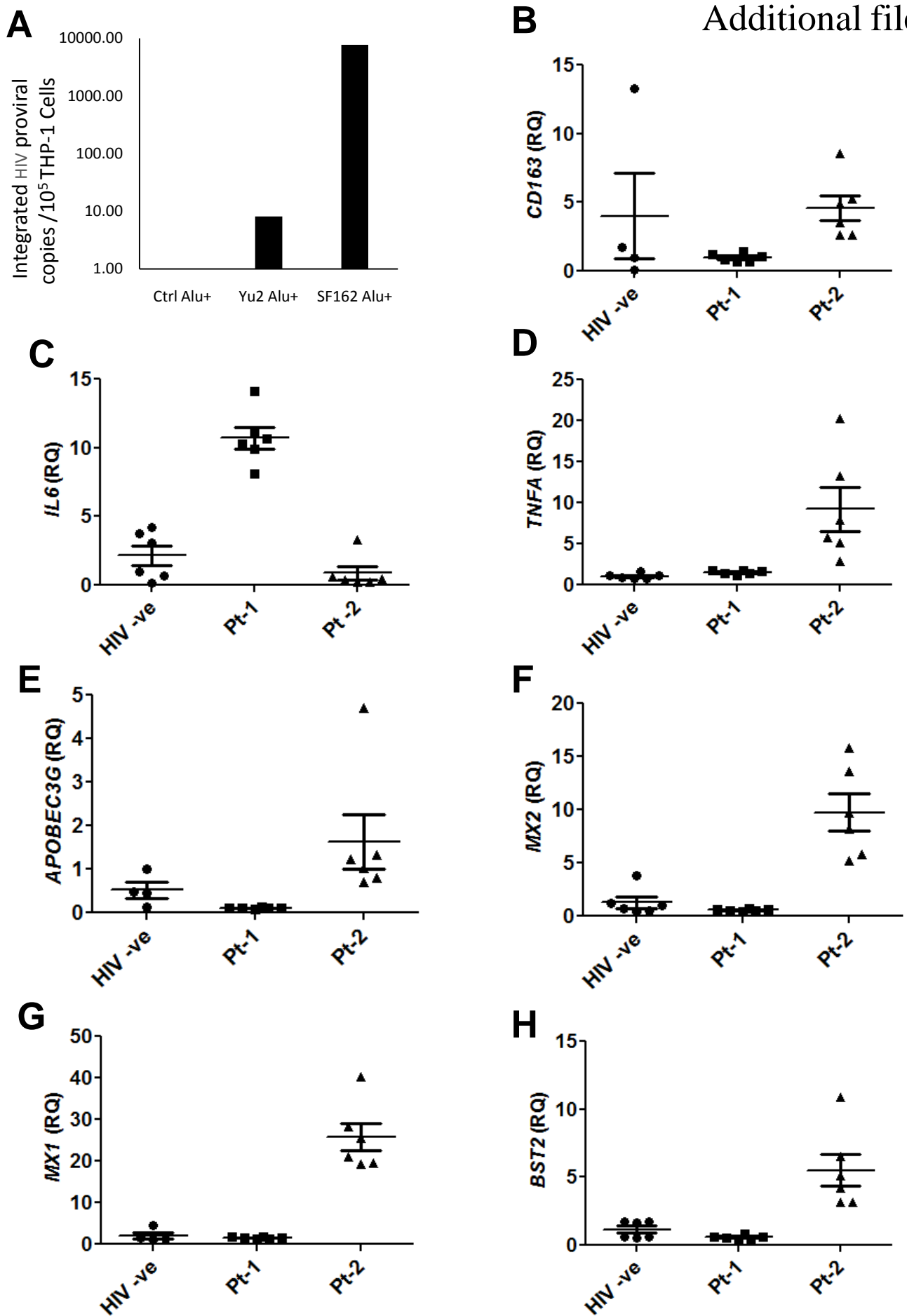

Supplement: Supplementary file 4 — Additional file 4. Differentiated THP-1 cells were infected with HIV-1 YU-2 and SF162. DNA was extracted from infected cells and used to establish the integration assay (A). Host genes expression from patients 1 and 2 compared to uninfected controls measured by quantitative RT-PCR for, (B) CD163, (C) IL6, (D) TNFA, (E) APOBEC3G, (F) MX2, (G) MX1 and (H) BST2. [file 12977_2017_370_MOESM4_ESM.pdf]

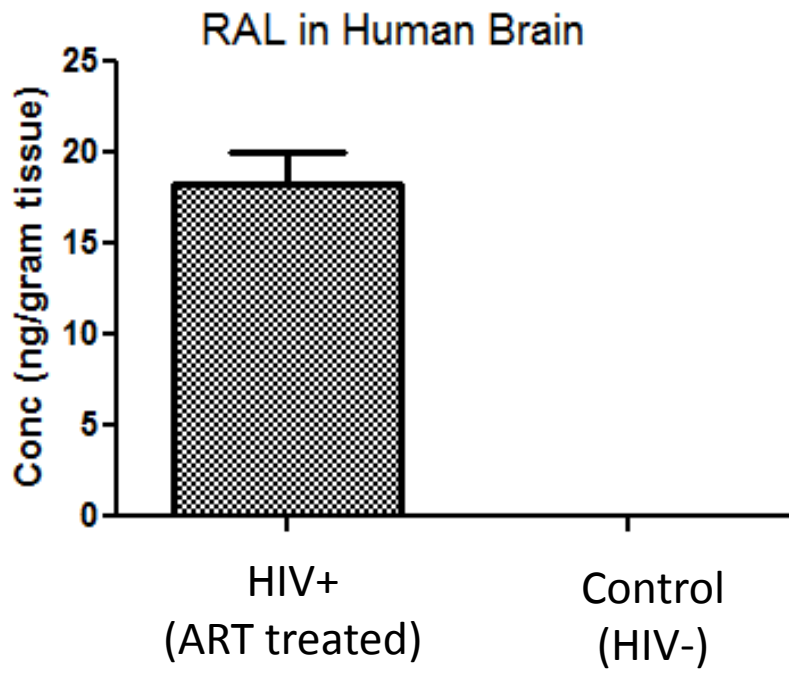

Supplement: Supplementary file 5 — Additional file 5. Brain tissue (70–100 mg) from different anatomic regions from patient 1 and uninfected controls were used to measure RAL concentration by HPLC-MS. [file 12977_2017_370_MOESM5_ESM.pdf]
